# Bioinspiration as a method of problem‐based STEM education: A case study with a class structured around the COVID‐19 crisis

**DOI:** 10.1002/ece3.8044

**Published:** 2021-08-25

**Authors:** Emilie C. Snell‐Rood, Dimitri Smirnoff, Hunter Cantrell, Kaila Chapman, Elizabeth Kirscht, Elizabeth Stretch

**Affiliations:** ^1^ Department of Ecology, Evolution and Behavior University of Minnesota‐Twin Cities Saint Paul Minnesota USA; ^2^ Department of Curriculum and Instruction Saint Paul Minnesota USA

**Keywords:** biomimetics, creativity, problem‐based learning, SARS‐CoV‐2

## Abstract

Bioinspiration is a promising lens for biology instruction as it allows the instructor to focus on current issues, such as the COVID‐19 pandemic. From social distancing to oxygen stress, organisms have been tackling pandemic‐related problems for millions of years. What can we learn from such diverse adaptations in our own applications? This review uses a seminar course on the COVID‐19 crisis to illustrate bioinspiration as an approach to teaching biology content. At the start of the class, students mind‐mapped the entire problem; this range of subproblems was used to structure the biology content throughout the entire class. Students came to individual classes with a brainstormed list of biological systems that could serve as inspiration for a particular problem (e.g., absorptive leaves in response to the problem of toilet paper shortages). After exploration of relevant biology content, discussion returned to the focal problem. Students dug deeper into the literature in a group project on mask design and biological systems relevant to filtration and transparency. This class structure was an engaging way for students to learn principles from ecology, evolution, behavior, and physiology. Challenges with this course design revolved around the interdisciplinary and creative nature of the structure; for instance, the knowledge of the participants was often stretched by engineering details. While the present class was focused on the COVID‐19 crisis, a course structured through a bioinspired approach can be applied to other focal problems, or subject areas, giving instructors a powerful method to deliver interdisciplinary content in an integrated and inquiry‐driven way.

## Graphical abstract

From social distancing to oxygen stress, organisms have been tackling pandemic‐related problems for millions of years. What can we learn from such diverse adaptations in our own applications? We use a seminar course on the COVID‐19 crisis to illustrate bioinspiration as an approach to teaching biology content.

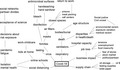

## 1 INTRODUCTION

Over the last two decades, there have been increasing calls for science education to use problem‐ and inquiry‐based approaches (Hmelo‐Silver, 2004; Norman & Schmidt, 1992; Schmidt et al., 2011), while also integrating across the many scientific disciplines that are often siloed (Brewer & Smith, 2011; Kelley & Knowles, 2016; Stohlmann et al., 2012). These interdisciplinary approaches more effectively motivate students to learn science concepts, relative to historical “content‐for‐content sake” approaches (Mustafa et al., 2016). In addition, active learning approaches tend to effectively train students in critical thinking and science process skills such as literature review and information synthesis (Coil et al., 2010; McConnell et al., 2003; Setiawaty et al., 2018; Walker, 2003). However, within higher education, the field is still exploring a range of ways to implement problem‐, inquiry‐, and integration‐based approaches, as teaching core content is often more straightforward, particularly in the rapid switch to online teaching during the COVID‐19 pandemic. In this manuscript, we illustrate how “bioinspiration” can be used as a general approach to accomplish all of these goals by structuring a class around topical problems.

Bioinspiration (or biomimetics) is a problem‐solving approach that looks to diverse biological traits for inspiration in human applications; organisms have been “solving” problems analogous to ours over evolutionary time (Bar‐Cohen, 2005; Bhushan, 2009). Examples such as mini‐drones inspired by insect flight (Floreano & Wood, 2015; Phan & Park, 2020), natural product discovery (Dias et al., 2012; Ratcliffe et al., 2011), and the naked mole rat as a model in cancer biology (Buffenstein, 2008; Zhao et al., 2019) show that we have much to learn from the diverse adaptations of the >10 million species on earth. Bioinspired approaches are increasingly common in engineering, chemistry, and architecture and are potentially applicable to any field or problem (Snell‐Rood, 2016; Wanieck et al., 2017). Biomimetic approaches greatly expand the space of potential solutions explored when problem‐solving (Graeff et al., 2018, 2019; Vincent et al., 2006), and design solutions are often improved by taking inspiration from biological traits (Jacobs et al., 2014; Kennedy & Marting, 2016). The nature of biomimetics also makes it a useful way to frame biology courses, as it is a problem‐based approach to biology content (Gardner, 2012; Stevens et al., 2019). Educators can use a biomimetic approach to structure inquiry and content around the most pressing issue of the day. Problems are abstracted into functional concerns, and students explore biological traits and systems that have explored similar functional space over evolutionary time (Fayemi et al., 2017).

Here, we detail a case study of using a bioinspired approach to structure an undergraduate biology course. Specifically, this case study focuses on a summer 2020 class centered on one of the most pressing issues at the time—the COVID‐19/SARS‐CoV‐2 pandemic. Our class “Biomimetic approaches to pandemics” explored how bioinspired approaches could broaden the solution space explored when solving problems related to COVID‐19. The basic format of the class worked well in an online format and was engaging for students. This review presents highlights of our process, to demonstrate the utility of a biomimetics approach to structuring a biology class, resulting in an integrated, interdisciplinary, and problem‐based STEM course. We first give an overview of our initial exploration of the COVID‐19 crisis through a problem‐mapping exercise that we used to structure the entire course. Second, we walk through two focal problems—respiratory distress and cooperation with public health measures—to illustrate how different problems can be matched to the biology content one wishes to deliver. Third, we go deeper into the problem we chose to explore for our class project (mask design). In this review, we hope to not only illustrate our educational process but also provide some detail in problem areas where we feel we made promising insights. While we are not virologists or epidemiologists, we hope that some of these ideas could serve as inspiration for creative approaches to solving pandemic‐related problems during this or future outbreaks. We conclude this review by discussing some of the broader lessons and challenges in a bioinspired teaching format and areas for future work.

## 2 ENGAGING STUDENTS AND STRUCTURING THE CLASS: MAPPING THE PANDEMIC PROBLEM SPACE

**FIGURE 1:**
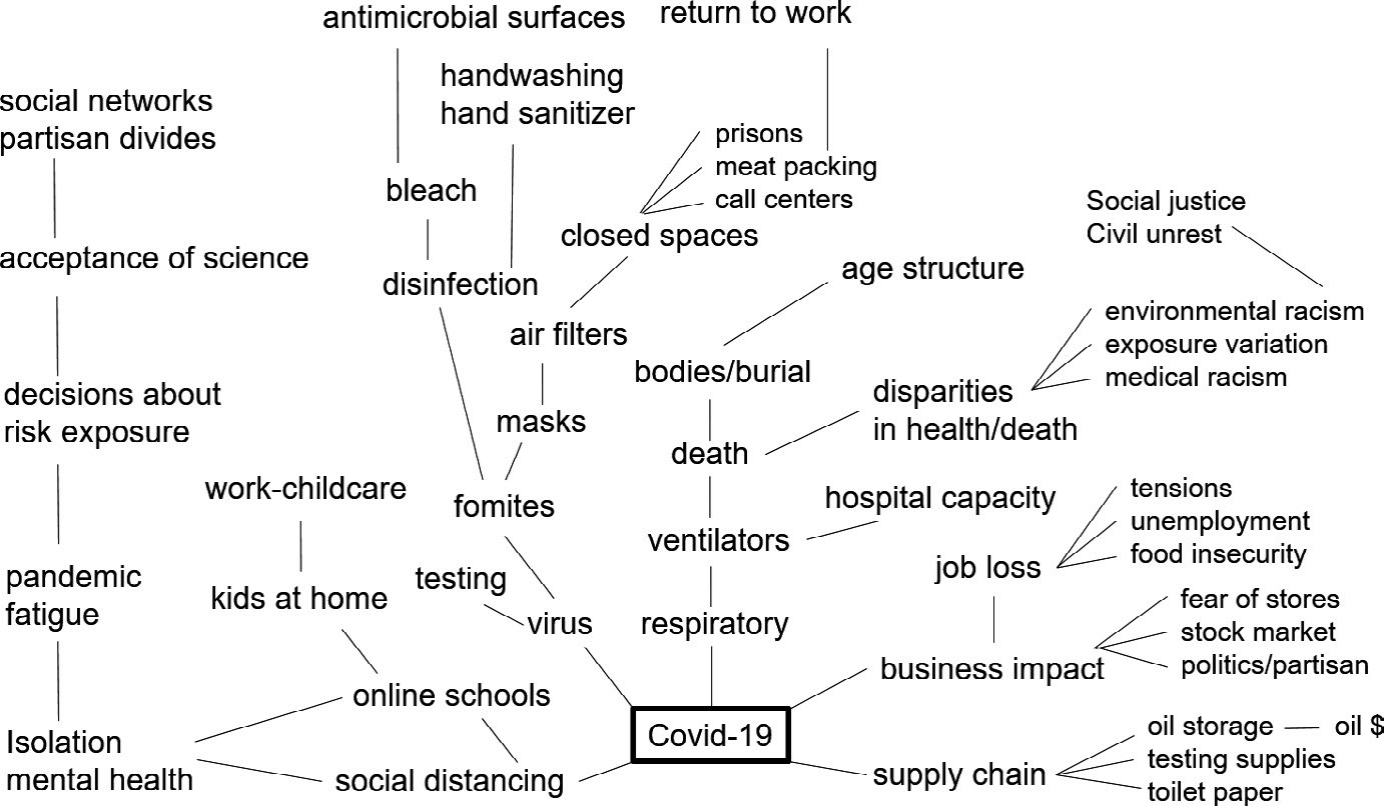
Sample mind mapping of the COVID‐19 problem. Sample “mind map” of the COVID‐19 problem. Individual lines connect ideas in a free‐flowing brainstorming session (e.g., “X makes me think of Y”), rather than any particular causal structure. This mind mapping helps to identify the many subproblems within a larger problem. In comparing the problem maps from different students, we also see how individual experiences shape the perception of high priority problems

We started our class by “mind mapping” the COVID‐19 pandemic (Davies, 2011; Edwards & Cooper, 2010), with each student taking a visual approach to exploring how the central problem of the class connects to dozens of other problems (Figure 1). The students recognized that while the COVID‐19 pandemic stems from a single‐stranded RNA virus (SARS‐CoV‐2), it extends into almost every space of science, technology, and society. The COVID‐19 pandemic encompasses problems related to addressing the disease in patients, including ventilators and antiviral medicines, and those related to disease spread, such as disinfectants, and social distancing. There are problems related to supply chain disruptions in the face of lock‐down, economic collapse in response to job loss and future unknowns, and problems related to childcare, mental health, and anxiety (Figure 1). This problem space is vast, which represents an opportunity for students to choose problems of individual interest, and for instructors to choose motivating problems matched to relevant course content (Table 1).

**TABLE 1:** Overview of course structure

Focal COVID problem	Biological functions explored	Biological Concepts and disciplines studied
Making effective masks	Filtration and transparency	Adaptation by natural selection, tradeoffs, nonadaptive traits, nature of biological traits
Toilet paper	Liquid absorption	Selection in ecological context, biomes, abiotic and biotic variables, habitat selection, scale, and function
Respiratory distress	Oxygen stress/anoxia	Macroevolution and convergence, independent origins, evolutionary constraints, and contingencies
Inflammation	Immune responses	Diversity of physiological adaptations, arms races, and host‐parasite coevolution
Antiviral medications	Viral defenses	Gene and protein function, understanding molecular variation, exploring chemical space
Mental health, anxiety	Risk assessment	Behavior, cue response systems, information, and assessment of environment in animal behavior
Following public health measures	Cooperation and altruism	Frequency dependence, game theory, payoff matrices, evolution of behavior
Economic collapse	Ecosystem stability	Ecosystems, networks of interactions, robustness, and resilience in systems biology

Note

We used our problem mind mapping exercise (Figure 1) to generate a list of problems related to COVID‐19. Aspects of these problems were distilled to biological “functions” that allowed analogies with biological traits and systems. Some problems were better matches to relevant biological content, which determined the presentation order of problems throughout the course. For example, thinking about trait form, function, and morphology is particularly suited to concepts related to natural selection and ecology, so related problems (e.g., building a mask or a filter) were placed at the start of the course.

Mind mapping the pandemic problem space revealed individual biases in how people explore problems. Each individual in the class mapped the COVID‐19 problem space separately and then compared notes (see Appendices S1). Individual mind maps look very different depending on each person's experience with the problem: People with small children in their immediate social network highlighted many of the challenges of childcare and working from home, while those who had to continue in‐person work highlighted problems with enforcing mask mandates in public spaces.

Iterative mind mapping proved useful for further exploring individual problems. Each individual problem could be broken down to individual components that might be more amenable to problem‐solving. For instance, “toilet paper shortages” was mapped further to liquid absorption, supply chain issues, hoarding and reselling, alternate materials, and questions about why we use toilet paper in the first place. Sequential mind mapping illustrates the broader importance of problem analysis in bioinspired approaches, both to determine possible intervention points and to find a range of possible biological analogs (Fayemi et al., 2017; Wanieck et al., 2017).

**FIGURE 2:**
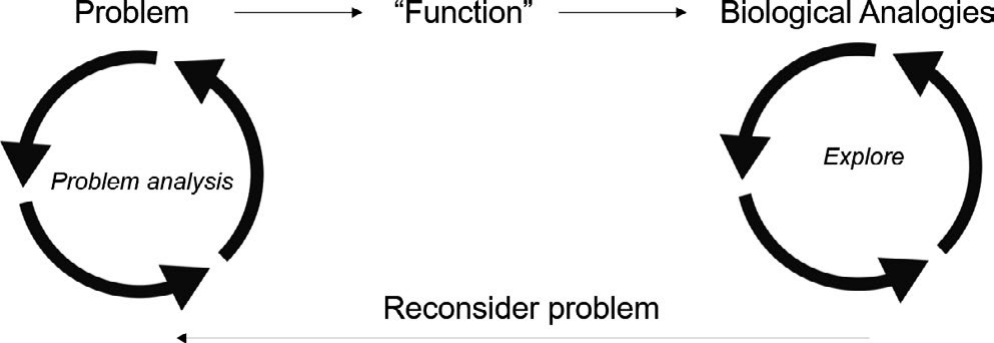
Overview of structure of each individual class. In each class, we spent some time discussing the relevant problem and extracting relevant “functions” (see Table 1) before exploring relevant biology and then reconsidering the problem at the end of the class. Did the basic biology offer a new perspective on the problem? This framework was developed through ideas from both the biomimetic process (Fayemi et al., 2017) and creative exploration (Stretch & Roehrig, 2021)

In subsequent classes, we used our overall exploration of the COVID‐19 crisis as a lens to explore a range of subfields in biology (Table 1). Each class explored both the pandemic and relevant biological concepts through a bioinspired approach. Individual classes focused on a problem related to COVID‐19, as inspired by our mind maps. Before class, students start by brainstorming around a biology “function” that allows analogies with the focal problem, for instance, using the *AskNature* database to find systems that filter particles or resist infection (Deldin & Schuknecht, 2014, Appendices S1, S2). Class time is spent exploring these systems, and the relevant biology (Figure 2, Appendices S1, S2), moving through major subdisciplines of biology, from evolution and ecology to physiology and behavior (see Table 1). We next walk through some of the material from the course to illustrate this process.

## 3 USING DIFFERENT COVID‐19 PROBLEMS TO EXPLORE DIFFERENT AREAS OF BIOLOGY

When using a bioinspired approach to a course, the instructor can choose the focal problems that best illustrate the biological concepts they wish to deliver (Table 1). To illustrate this idea, we walk through the types of content matched to two of the focal problems covered in the class—respiratory distress and encouraging the public to conform to public health measures. These examples illustrate the overall approach to each individual class: analyze the focal problem, articulate associated functions that translate to biological analogies, explore relevant biology, and finally reconsider the problem (Figure 2).

### 3.1 Dealing with low oxygen and inflammation—exploring physiology

To use a bioinspired approach, one first needs to explore the focal problem in enough detail to match the problem to biological analogies. The idea of “function” provides a bridge between biology and design fields such as engineering (Cohen et al., 2014; Lepora et al., 2013; Norberg, 2002; Thielen et al., 2013; Vincent et al., 2006). What “function” are you trying to tackle in a given application and what biological systems have evolved, over millions of years, to cope with a similar functional challenge? One problem may be associated with several different functional concerns, each of which may suggest different biological systems for study (see Appendices S1, S2 for examples). For example, SARS‐CoV‐2 infection comes with a range of multiple medical challenges, one of the primary being low blood oxygen, sometimes presenting without behavioral indicators of oxygen stress (“silent hypoxemia” or hypoxia, Tobin et al., 2020; Wilkerson et al., 2020). This observation suggests considering diverse organismal adaptations to cope with low oxygen conditions, whether due to variation in elevation, water temperature, or physiological changes such as hibernation. Students came to class with their own ideas, often by using the online database *AskNature* (Deldin & Schuknecht, 2014), and we used class time to explore concepts around evolutionary physiology. For example, with respect to low oxygen environments, many animals cope through evolutionary and physiological changes that affect how hemoglobin in red blood cells captures oxygen. For example, some fish survive low oxygen conditions with increased hemoglobin binding efficiency (Nilsson & Renshaw, 2004), with a greater diversity of hemoglobins in species in more variable environments (Baalsrud et al., 2017). There is a huge diversity of hemoglobins in birds as well, representing independent adaptation to high elevation through increased oxygen binding (Storz, 2016). Interestingly, human populations at high elevations also exhibit diverse adaptations to low oxygen conditions (Beall, 2007; Storz, 2010) and have decreased susceptibility to SARS‐CoV‐2 (Arias‐Reyes et al., 2020). After considering the diversity of physiological mechanisms to deal with low oxygen, we considered how some examples might be more promising than other with respect to the focal problem. For instance, some companies are in the early stages of blood transfusion treatments using unusual hemoglobins to treat COVID‐19 (e.g., from a marine worm, Garraud, 2020; Le Gall et al., 2014), similar to related treatments that pack red blood cells or saturate cells with oxygen (Geier & Geier, 2020). However, use of hemoglobins adapted to low oxygen environments may be limited by the ability of these hemoglobins to release oxygen to stressed tissues. Adaptations in human populations seemed to give more immediately feasible ideas for COVID‐19 treatments, such as encouraging physiological acclimation to low oxygen conditions (e.g., altitude training, Mairbaurl et al., 1986; Saunders et al., 2009; Subudhi et al., 2014) or possible use of phytochemical drugs (Biondich & Joslin, 2015; Chiang et al., 2015).

If an instructor wishes to highlight more molecular concepts in physiology, rather than evolutionary concepts, the relevant examples and content exploration can be adjusted. For instance, in one class, we delved into concepts around molecular signaling pathways by talking about naked mole rats. Some subterranean rodents such as naked and blind mole rats can survive in 5% oxygen conditions for hours, through a number of adaptations, from reductions in metabolism and shifts to anaerobic glycolysis, to induction of unconsciousness (Park et al., 2017). A key adaptation in these species involves changes to the Nrf2 signaling pathway, which is a key regulator of stress responses in mammals. Naked mole rats and other relatively longer‐lived species have higher Nrf2 signaling, generally achieved through reduced degradation of Nrf2 (Lewis et al., 2015; Schmidt et al., 2016). After working to understand the details of the signaling pathways and physiology, we returned to the problem—what, if anything, do such observations suggest for SARS‐CoV‐2 interventions? Targeting the upregulation of this pathway could be feasible and, indeed, has already been suggested as a potential therapeutic target (McCord et al., 2020). A number of plant chemicals are known stimulators of Nrf2 signaling, with beneficial effects when taken at low doses (Hybertson et al., 2019; Juge et al., 2007; Mattson & Cheng, 2006); interestingly, some act through reduced degradation of Nrf2, analogous to adaptations in naked mole rats (Zhang et al., 2019).

Giving students a degree of agency and choice in structuring a course increases engagement (Holmes et al., 2020; Luo et al., 2019; Schmidt et al., 2018). In the present course, student–instructor discussions generated lists of possible biological systems to explore for a given problem. For subsequent discussions, students gave input on which ideas they found the most feasible or exciting for future exploration and development, which determined the direction of the class and the content. For example, in discussing the physiological stress of inflammation, the idea of bat immunity came up as a possible system. The next class was designated as a deeper dive into bat immune adaptations, as bats harbor many coronaviruses (Banerjee et al., 2019) but rarely show external symptoms in response to these diseases (Baker et al., 2013; Hayman, 2019; Mandl et al., 2018). The bat immune system has a constitutively expressed innate immune response (the interferon pathway, Banerjee et al., 2020; Brook et al., 2020), but suppresses inflammation, which can be costly (Ahn et al., 2019). This exploration into bat immunity was student‐driven, as it was not included in the original syllabus, and ended up highlighting the importance of depressing the inflammation response in treating COVID‐19 (Tay et al., 2020), similar to how bats repress pro‐inflammatory cytokines (Banerjee et al., 2020; Leyfman et al., 2020; Schett et al., 2020).

### 3.2 Encouraging cooperation with public health policies—exploring behavior

In some cases, the mapping between a focal problem, the associated function, and a biological analogy is straightforward, for instance in considering physiological adaptations to low oxygen. However, the links to biological analogies may be less obvious for other problems—in such cases, we need to more thoroughly dissect the problem, as illustrated by our class on cooperation with public health recommendations. Many public health measures during pandemics require some degree of self‐sacrifice for the greater social good. For instance, wearing masks comes with some personal discomfort, but we need a critical mass of the public to be wearing them (e.g., cloth masks with 60% efficacy require 60% of the public to wear masks to contain the virus, Tian et al., 2020). How do we encourage cooperation with these policies? Here, we can turn the problem into a cooperation “game” that allows analogies to behavioral ecology. Classic game theory highlights the evolutionary challenges of altruistic strategies: The temptation to be selfish or “defect” results in “cheaters” that benefit when they are rare, but the entire system begins to suffer as they increase in frequency (Doebeli & Hauert, 2005; Fehr & Rockenbach, 2004; Perc & Szolnoki, 2010; Smith, 1979). The “payoff matrix” affects the relative costs and benefits of cooperation versus more selfish strategies (Doebeli & Hauert, 2005), but dynamics change as players in a game meet multiple times and make choices about where to hang out (Archetti et al., 2011; Perc & Szolnoki, 2010). We used this broad approach to explore whether studies of the ecology and evolution of cooperation and mutualism (West et al., 2007) could offer novel insights for human cooperation with public health measures.

We turned our attention to exploring examples of cooperation and mutualism where unrelated individuals cooperate; such examples are more analogous to the focal problem of cooperating with mask mandates than eusocial ants rallying around their maternal queen. We explored in more detail one particular example, the mutualistic relationship between cleaner fish and its reef fish clients. Cleaner fish pick ectoparasites off client fish, but actually prefer to eat client scales or mucous, which is costly to clients (Grutter & Bshary, 2003). Clients encourage cooperative behavior by altering both the associated benefits and costs, for instance, ending interactions early with uncooperative cleaners, often aggressively chasing them away (a cost or punishment, Bshary & Grutter, 2002). Clients will also stay longer for more cooperative cleaner fish (a reward or benefit, Gingins & Bshary, 2015). The cleaner fish system also highlights another mechanism of cooperation: reciprocal altruism, which emerges when identifiable individuals have repeated interactions. Reciprocal altruism has strong support across theory and many plant and animal systems (Kiers et al., 2011; Raihani & Bshary, 2011; West et al., 2007). In client fish, this plays out both directly and indirectly. Client fish are more likely to return to a cleaning location with cooperative cleaner fish, but are more likely to change cleaners if they had previously been cheated or ignored (Bshary & Schaffer, 2002). This cleaner fish system also shows evidence of “indirect” reciprocity, where individuals are more likely to cooperate with individuals with a cooperative reputation, and individuals are thus sensitive to how a current action affects their reputation: Cleaner fish are more likely to cooperate when there is an audience (Pinto et al., 2011) and adjust cheating depending on social context, “managing” their reputation.

After an exploration of examples of cooperation in unrelated individuals, we turned to discussing how such biology could offer insights or creative ideas for the focal problem—can we get people to wear masks in stores? We first considered how we might encourage cooperation by altering the costs and benefits of the payoff matrix for one interaction between participants. Here, we noted how some theory suggests that rewards for cooperation may have been more important in the evolution of cooperation than often appreciated (Gingins & Bshary, 2015; Weyl et al., 2010), suggesting that a focus on punishing cheaters (Douglas, 2008; Raihani et al., 2012) may be less effective in applications to promote cooperation. These studies led to discussions how we might increase rewards for mask‐wearing, such as government incentives to reward masks in stores (e.g., game theory and incentives in vaccinations, Chapman et al., 2012), or promoting masks as fashionable self‐expressions (e.g., fitness benefits of “trends,” Kokko et al., 2007). We then turned to thinking about interventions that might encourage cooperation related to reciprocity. Here we considered what kinds of social behavior manipulation are truly ethical: If individuals are more likely to be cooperative if their identity is known, do we start requiring individual identification to be displayed in public spaces? The students responded: “probably not!” Do we build on studies showing increased cooperation when individuals feel they are being watched, by affixing staring eyes to grocery store walls (Bateson et al., 2006)? Again, the students responded “probably not!” However, this discussion brings up complex debates about the ethics of subtle behavioral manipulations through cognitive biases which often seem ethical in the health realm (“nudges,” Marteau et al., 2012) but less so in the advertising realm, even if they are frequently used in marketing (Jolls et al., 1998; Kahneman, 2003; Wilkinson, 2013). Although these latter examples may be less ethical or feasible, this line of questioning highlights how exploring a human challenge through a biological lens can prompt novel thinking.

## 4 CLASS PROJECT: DIGGING DEEPER INTO MASKS, AIR FILTRATION, AND MORPHOLOGICAL ADAPTATIONS

A major goal of this course was to give students an opportunity to dig deeper into biological material, finding relevant literature, critically evaluating research findings, and integrating and applying knowledge to new situations. We accomplished this goal through a class project, the structure of which we worked out through group discussions. Students had agency in not only the format of the project, but also the focal problem—two weeks into the course, they voted on the topic they were most interested in exploring in‐depth, choosing mask design and air filtration. Airborne transmission is a key component of the rapid spread of SARS‐CoV‐2 (Prather et al., 2020). Viral particles suspended in respiratory droplets result in increased rates of transmission in indoor spaces (Allen & Marr, 2020). Thus, wearing masks and increasing ventilation are often highly effective control measures (Cheng et al., 2020; Wang, Tian, et al., 2020). Coronaviruses are very small (on the order of 0.1 µm in diameter), and many materials that effectively block individual viral particles also reduce airflow, limiting their utility in masks (Davies et al., 2013; Zangmeister et al., 2020). However, many materials can block larger respiratory droplets (5–10 µm), which can reduce disease spread (Bourouiba, 2020; Stadnytskyi et al., 2020). Thus, cloth masks are recommended for broad use when more effective N95 masks are not available. Filtration systems that purify indoor air (e.g., stand‐alone or HVAC HEPA filters) can filter out viral particles using a wider range of materials that are not necessarily breathable (Zhao et al., 2020). For instance, the use of HEPA filtration in hospitals can cleanse the air of SARS‐CoV‐2 particles (McDonald, 2020; Phu et al., 2020). For our course‐long project, we explored whether bioinspiration could offer ideas for the design of more effective, efficient, or easy‐to‐use masks or air filtration systems. We focused on two functions—filtration and transparency.

### 4.1 Filtration systems

We first explored a range of ways in which “filtration” applies to biological systems and traits. Many animals use filter‐feeding to separate food particles from the surrounding air or water. In other cases, biological materials that have evolved in one context (e.g., plant fibers) can be used as filters in human applications—in other words, filtration is a function in human applications rather than the evolved function of that trait (“bioutilization,” Montana Hoyos & Fiorentino, 2016). Throughout the project, we went through an iterated series of exploration of the biology, starting the first week with an overview of filtration systems, and then progressively diving deeper as students researched specific systems or traits (see examples in Appendices S1). During the last week of class, we invited an engineer and fluid mechanic to the course to give feedback on the ideas (recognized in acknowledgements). Here, we walk through some of the filter‐feeding systems we explored, and how this deeper research gave insights into the design of masks or air purifiers.

While we initially brainstormed a long list of filter feeders, reading papers on flamingos, and even considering black fly larvae, we soon focused on marine filter feeders due to their incredible diversity, including filtration systems matched to very small particles. Marine organisms show a wide range of filter‐feeding mechanisms that span all of the primary mechanisms of filtration shown in human‐engineered filters (and more). Filter‐feeding organisms vary in size from microscopic to the largest animals on earth (baleen whales). Animal filtration systems selectively exclude or include particles down to the micron level, meaning there are potential applications for blocking the aerosol particles relevant in COVID‐19 transmission, in addition to potentially the viral particles themselves. How are they filtering particles? Students divided their research efforts across species that used different filtration mechanisms. Many marine species that have been assumed to use sieve‐based filtration actually rely on a combination of filtration mechanisms, including crossflow filtration (i.e., fluid flow parallel to the filter, Conley et al., 2018). Some marine tunicates (appendicularians) have a two‐stage filtration system that relies on morphological shape, behavior, and properties of the mucous mesh to filter and concentrate particles as small as viruses, that are important components of their diet (Conley, Gemmell, et al., 2018; Lawrence et al., 2018). Tunicates vary tremendously in the structure and morphology of their filtration systems and mucous nets with functional implications for filtration rate, and the size and shape of filtered particles size (Sutherland et al, 2010; Conley & Sutherland, 2017). While some of the filtration mechanisms overlap with those used in industrial filters, in many cases, aspects of the biological filters are unique; for instance, filter movements can result in selective removal of some particles, a form of filter self‐cleaning (Conley, Gemmell, et al., 2018). Outside of tunicates, other marine organisms also filter in unexpected ways. Manta rays exhibit a novel mechanism termed “ricochet separation” that separates particles based on size and prevents filter clogging (Divi et al., 2018), a discovery which has inspired novel industrial filters capable of separating nanoparticles (Wang, Xu, et al., 2020). The diversity of marine filtration systems could potentially inspire creative new directions in the design of more effective or longer lasting facemasks or air purifiers; we were particularly intrigued by the systems that used combinations of mechanisms, including behavioral manipulations of the filter.

### 4.2 Mask transparency

In addition to exploring the primary function of masks (filtration), we also identified other challenges associated with masks, such as transparency (see examples in Appendices S1). People with hearing loss and social communication disorders can have difficulties communicating when they cannot see the mouth movements of the others in the conversation (Chodosh et al., 2020; Corey et al., 2020), an issue advocated by a student in the course from the Speech‐Language‐Hearing Sciences MA program. Creating transparent marks is challenging because most existing transparent materials, such as glass and plastic, are not breathable and often fog. We considered whether bioinspired approaches could be used to develop novel transparent textiles, specifically a breathable, transparent material that is also an effective filter.

**FIGURE 3:**
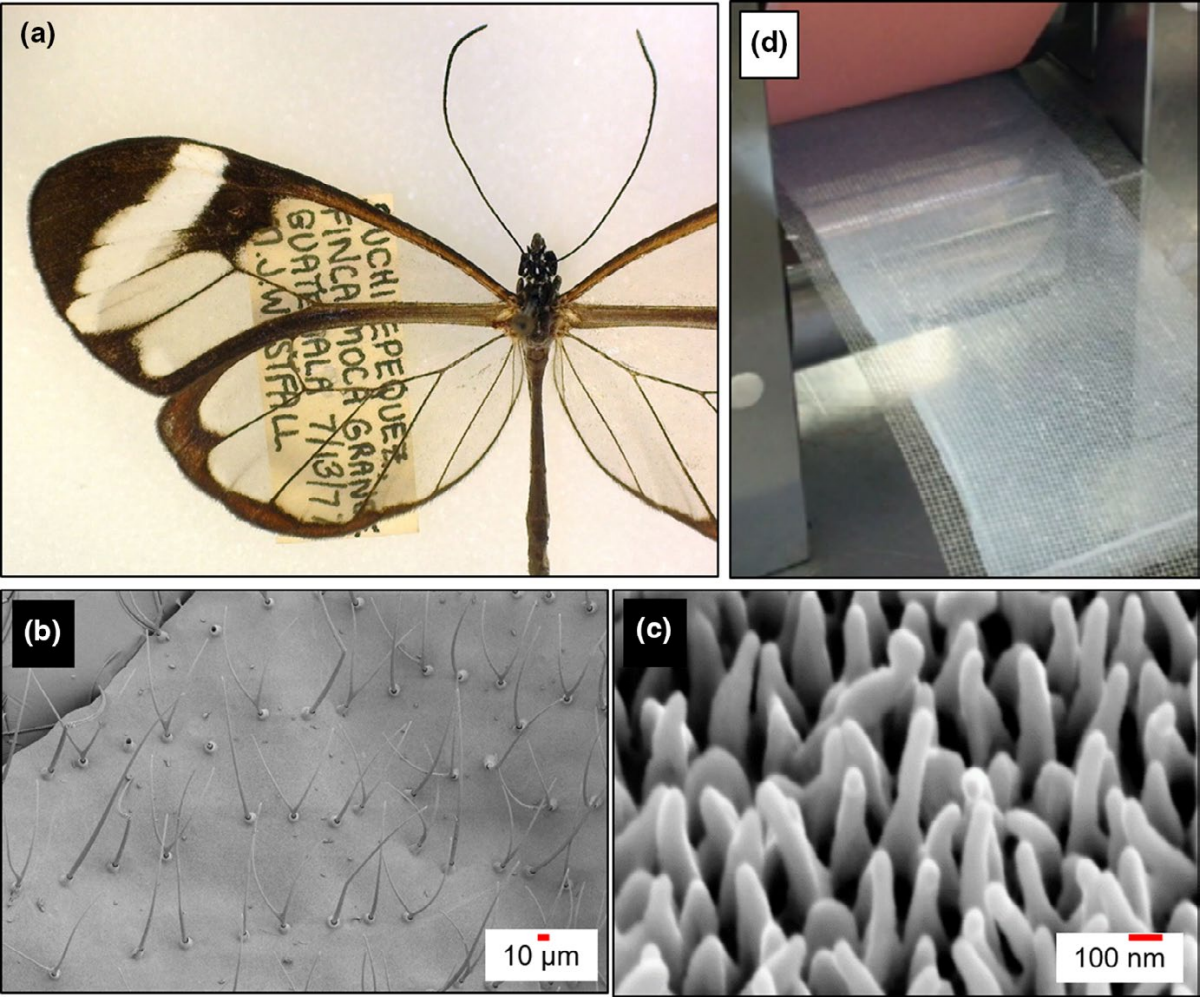
The glasswing butterfly as inspiration for transparent face masks. (a) The glasswing butterfly (*Greta oto*) relies on transparent wings for camouflage in rainforests of Central and South America. This transparency stems from antireflective surfaces on their wing cuticle characterized by small hairs (b) interspersed with densely packed nanopillars (c). Electrospinning, which has already been used to synthesize semi‐transparent filters (d), could potentially be used to develop transparent face masks. Image credits: (a) Image courtesy of Andrei Sourakov; (b, c) SEMs courtesy of Radwan Siddique; (d) reprinted (adapted) with permission from (Xu et al. 2016 Nano Lett). Copyright (2016) American Chemical SOCIETY

Butterflies, and insects more generally, have evolved transparent wings independently multiple times, often with diverse underlying mechanisms (Dushkina et al., 2017; Watson et al., 2017). The wings of most butterflies and moths are semi‐translucent when wing scales are removed, but in some cases, there has been selection on transparency to facilitate effective camouflage (Figure 3a, Gomez et al., 2021; Johnsen, 2001; McClure et al., 2019). Many of these wing structures are additionally hydrophobic, another desired characteristic in mask design (El‐Atab et al., 2020; Wanasekara & Chalivendra, 2011). Our research honed in on one butterfly species in particular. The wings of the glasswing butterfly are both antireflective and translucent because of randomly dispersed pillar‐shaped nanostructures (about 4 µm in width) on the wing surface (Figure 3c, Pomerantz et al., 2021; Siddique et al., 2015). Creating similarly sized and shaped structures on glass reduces reflectivity and increases transparency (Diao et al., 2016; Sourakov & Al‐Obeidi, 2019), but is it possible to use an analogous design to make a breathable fabric more opaque? “Electrospinning” can be used to create fibers as small as 2 μm in width and has been applied to breathable fibrous materials (Frey, 2008; Xue et al., 2019). Indeed, electrospinning has been used to create translucent air filtering materials (Figure 3d; He et al., 2020; Xu et al., 2016), suggesting inspiration from butterflies could improve on existing designs to create bioinspired transparent face masks (Eadie & Ghosh, 2011). These ideas, some of our most promising in the end, emerged out of six successive class discussions, literature research in preclass assignments, and input from engineers who visited the class (and suggested the electrospinning approach).

## 5 MOVING FORWARD: APPLYING PEDAGOGICAL SUCCESSES AND ADDRESSING CHALLENGES

Over the last several decades, there have been increasing calls to revise undergraduate science education to be integrated across disciplines and focused on inquiry and problem‐solving (Aikens, 2020; Brewer & Smith, 2011; Norman & Schmidt, 1992; Suwono et al., 2018). In this paper, we illustrate one such approach—using biomimetics to tackle whatever problem is currently the most pressing to students. This approach is engaging because everyone (including the instructor) is often consumed with this problem. At the same time, this approach makes content from diverse fields, from fluid dynamics to molecular biology, immediately relevant. Students generally responded positively to the course, but several challenges were raised in this pilot iteration of the course (Table 2). We close by exploring several general challenges raised by the course and possible ways forward.

**TABLE 2:** *A sampling of student reactions to the course*, both immediately following the course (August 2020) and a year later (June 2021). Reactions were paraphrased from course evaluations and postcourse email follow‐ups

Strengths	Challenges
Engaging content, taught with relevance	Grading for brainstorming unclear
Environment for collaboration	Recruiting students to interdisciplinary classes
Encourages growth, exploration	Teaching engineering to nonmajors
Student agency, choice	Lack of laboratory or hands‐on experience
Prepared students to approach public health issues through bioinspiration	Interdisciplinary nature: what background is required versus reviewed
Small class size (4) optimal for discussion; space for free conversation	Larger class would have allowed for broader discussion, more perspectives

This course highlighted challenges around promoting creative exploration of ideas and biological systems. Individual classes were structured first around a problem discussion, then an exploration of ideas students brainstormed before class, followed by a deeper dive into some relevant biology content, and finally a return to the problem and evaluation of the biological ideas (Figure 2), guided by the creativity framework of Stretch and Roehrig (2021). Students were encouraged to consider and explore any and all ideas, even ones that might initially seem unfeasible or bizarre. This structure was successful because students were engaged—they came to class a creative list of biological systems they had brainstormed (see examples in Appendices S1), they were excited to share their ideas, and they had an immediate connection to the biology that followed. While this biology exploration engaged students’ creativity, it had two associated primary challenges. First, opening the full creative potential of our students stretched the content expertise of the instructors. Each week we explored a wide range of biological systems that came to mind, and while we could navigate much of the biology ourselves, we quickly learned that the primary limitation in moving these ideas into applications was relevant expertise. We needed input from biologists that were intimately familiar with the systems we were discussing, and designers, engineers, and doctors that could guide the next steps of moving an idea to an application. Future iterations of such a course would be improved by building teams of biologists and engineers (Graeff et al., 2018, 2019; Vincent et al., 2006), although guest appearances and email consultation by relevant experts sufficed for our 8‐week course.

Second, the assessment of the brainstorming exercises, which formed a major basis of the class grade, was challenging. To encourage a broad exploration, one needs to make mistakes, embrace failure, and not expect every idea to be perfect (the “exploration‐exploitation tradeoff, Cohen et al., 2007; Gopnik et al., 2017; Jansen et al., 2006; Stretch & Roehrig, 2021), so grades were assigned based only on assignment completion, not the quality of the ideas. However, it is possible that adding more detail to assignment prompts (specifically adding creativity) and developing rubrics for scoring certain aspects of creativity (e.g., number of taxonomic groups listed) could further promote creative biodiversity exploration. For instance, how do we push students beyond their initial biases in creative brainstorming as they often tend toward biological systems with which they are familiar? Further engagement with the STEM creativity field would be beneficial and is in progress for future iterations of the course (Forthmann et al., 2016; Silvia et al., 2008).

In summary, bioinspired approaches represent a promising method of structuring biology courses in a problem‐based way where disparate biology topics are integrated and immediately relevant. While there are a number of curricula designed around biomimetics (Stevens et al., 2019; Urmann, 2016; Wanieck et al. 2020), this case study illustrates how a bioinspired lens on a focal problem can be used to deliver biology content (Table 1). For instance, thinking about function of morphological traits maps onto discussions of adaptation and evolution, while medical applications mapped onto aspects of physiology and developmental genetics (Table 1). In navigating these topics, we also discussed designing biological experiments, reading primary literature, interpreting phylogenetic relationships, and a number of other topics. An instructor can structure content depending on the interests of students in the course and the specific focal problem tackled by the class. Several aspects of this course design were particularly effective in engaging students, including the problem‐based approach, giving students agency, and ample time for discussion and digging deeper into a project. In closing, we note that this small summer seminar of four participants provided a relatively low‐stakes way for the instructor to completely “flip” a class around bioinspired design, laying the groundwork for revision of other, larger enrollment courses, in a similar way and the collection of quantitative data to assess impact on students. Our current research focuses on a more thorough analysis of a large enrollment animal behavior course (180 students) restructured around bioinspired design, building on the methods and assignments developed in this COVID‐19‐focused course. Future research questions may consider how the bioinspired lens to class design may apply to undergraduate versus graduate student audiences, classes with and without a hands‐on laboratory component, and how building of teams with complementary expertise in biology and design may facilitate learning and problem‐solving.

## CONFLICTS OF INTEREST

The authors have no conflicts of interest.

## AUTHOR CONTRIBUTION

**Emilie C. Snell‐Rood:** Conceptualization (lead); Resources (lead); Visualization (lead); Writing‐original draft (lead); Writing‐review & editing (lead). **Dimitri Smirnoff:** Conceptualization (equal); Methodology (supporting); Visualization (supporting); Writing‐original draft (supporting); Writing‐review & editing (supporting). **Hunter Cantrell:** Conceptualization (supporting); Visualization (supporting); Writing‐original draft (supporting); Writing‐review & editing (supporting). **Kaila Chapman:** Conceptualization (supporting); Visualization (supporting); Writing‐original draft (supporting); Writing‐review & editing (supporting). **Elizabeth Kirscht:** Conceptualization (supporting); Visualization (supporting); Writing‐original draft (supporting); Writing‐review & editing (supporting). **Elizabeth Stretch:** Conceptualization (equal); Methodology (supporting).

## Supporting information

Appendix S1

Appendix S2

## Data Availability

All relevant additional materials can be found in the supplementary material. No data are associated with this review article.
