# Supplementary material for: Bioinspiration as a method of problem‐based STEM education: A case study with a class structured around the COVID‐19 crisis
Source: Ecol Evol. 2021 Aug 25;11(23):16374–86. doi: 10.1002/ece3.8044 (PMC8646331; doi:10.1002/ece3.8044)
Supplement: Supplementary file 1 — Appendix S1 [file ECE3-11-16374-s001.docx]

**Supplementary Material:**

**A. Overall class schedule**

| Date | Topic |
| --- | --- |
| June-9 | Motivation for class & intro to course structure |
| June-11 | Dissecting problems – problem map for covid-19 |
| June-16 | Form and function in biology  *Focal problem: filtration* |
| June-18 | Studying function and organismal biology  *(filtration continued)* |
| June-23 | Selective environments: ecology  *Focal problem: toilet paper and absorption* |
| June-25 | Evaluating feasibility in creative, collaborative explorations  (*toilet paper continued*) |
| June-30 | Macroevolution of traits and convergence of function  *Focal problem: oxygen stress* |
| July-2 | *Projects* |
| July-7 | Exploring the unknown in biological space  *Focal problem: antiviral medication* |
| July-9 | Diversification of physiological responses  *Focal problem: immunity and inflammation* |
| July-14 | Cue-response systems and the evolution of behavior  *Focal problem: mental health and anxiety* |
| July-16 | *Projects* |
| July-21 | Social interactions and game theory  *Focal problem: altruism and cooperation for public good* |
| July-23 | *Projects – interview with experts in engineering and fluid mechanics* |
| July-28 | Ecosystems and networks of interactions  *Focal problem: economic collapse* |
| July-30 | *Projects and next steps* |

**B. Example Class structure: (from Class 5, June 23)**

**Objectives and key concepts to take away from class**

- Learn about ecological environments that shape the evolution of biological traits
- Introduction to targets of selection versus functions as a product
- Understand abiotic variation – primary and secondary – and emergence of biomes
- Understand biotic variables and the importance of behavior influences microhabitat

**Background:** We are focusing on “toilet paper” and the function of “absorption” from our overall mind map. This lesson plan corresponds to the 5^th^ (of 16) classes. Starting early in the class with problems with “material” solutions (e.g., building a filtration system) are often a straightforward place to start in terms of exploring adaptations. Behavior, and system-related problems are easier to tackle later in the class after more basic concepts about evolution, ecology, and organismal biology have been worked through.

**Reading**: MacLean 1983 BioScience *Water transport by sandgrouse*

**Activities and structure**

1. **Pre-class assignments**:
   1. Make a mini-mind map for “toilet paper” – what additional problems or ideas come to mind around “toilet paper shortage”
   2. Brainstorm biological systems related to one focal function – in this case, the “absorption” capacity of toilet paper. List as many biological systems or traits that come to mind.
2. **Group discussion**: Review functions and sub-problems that come to mind around toilet paper shortages. Discuss a few additional ideas that came up on a google search (e.g., people used to use corn cobs!)
3. **Content**: clarify what we mean by “absorption” of liquid (capillary action, sorptivity of materials, other notes from fluids/physics)
4. **Discussion**: what biological systems of traits do you think of around “absorption.”
   1. Note that some of these are traits and properties of individual species (sponges), and some are byproducts of ecosystems (wetlands) or biological materials in new situations (cellulose fibers in paper towels). Absorption as a byproduct versus a target of natural selection.
5. **Discussion**: What environments do you think of when you think of selection on absorption as a trait? Environments with not enough water… and those with lots of water…
6. **Paper review and discussion** around a favorite example (sandgrouse feathers). Watch a few videos of sandgrouse behavior, review a few key figures from paper and go around and get individual student reactions from the reading.
7. **Content**: Ecology content… framed around how we can more thoroughly explore the types of environments that select for a particular function
   1. Review difference between abiotic and biotic factors
   2. Review geographic variation in primary abiotic factors (temperature, precip) and how this translates into biomes (see wikipedia article on biomes)
   3. Note minor abiotic variables may also matter (e.g., wind speed map)
8. **Discuss** how this knowledge might change how you are thinking about looking for biological systems for absorption – new ideas for terms in a literature search?
9. **Content**: Ecoregions (867 of them!) across 14 biomes: <https://en.wikipedia.org/wiki/List_of_terrestrial_ecoregions_(WWF)>
   1. **Activity**: navigate to one of these 867 ecoregions
   2. **Discuss**: why might you want to look at different geographic areas within an ecoregion?
10. **Content:** biotic variables
    1. Example: plants are also playing a role in water uptake
    2. Can we make geographic generalizations about biotic variables (E.g., variation in biodiversity worldwide?)
    3. Behavior and physiology structures how individuals experience variation
11. **Discuss:** where would you look for absorption based on discussion of these biotic axes? Would different deserts be complementary?
12. **Back to the problem** and bio-inspiration
    1. An example of bio-inspired fog harvesters (absorption from gas to liquid)
    2. Return to toilet paper and absorption
    3. **Activity:** go back to list of examples you came to class with…how would this change? How would your subsequent search and research change?

**C. Example assignments from students**

**1. Mind-map of COVID-19 Crisis (for class 2).** *Prompt for Assignment*: Use a mind-mapping approach to sketch out all of the problems you can possibly think of associated with the covid-19 pandemic. Treat this as a stream-of-consciousness. Take a picture or upload an image.

**Sample response:**


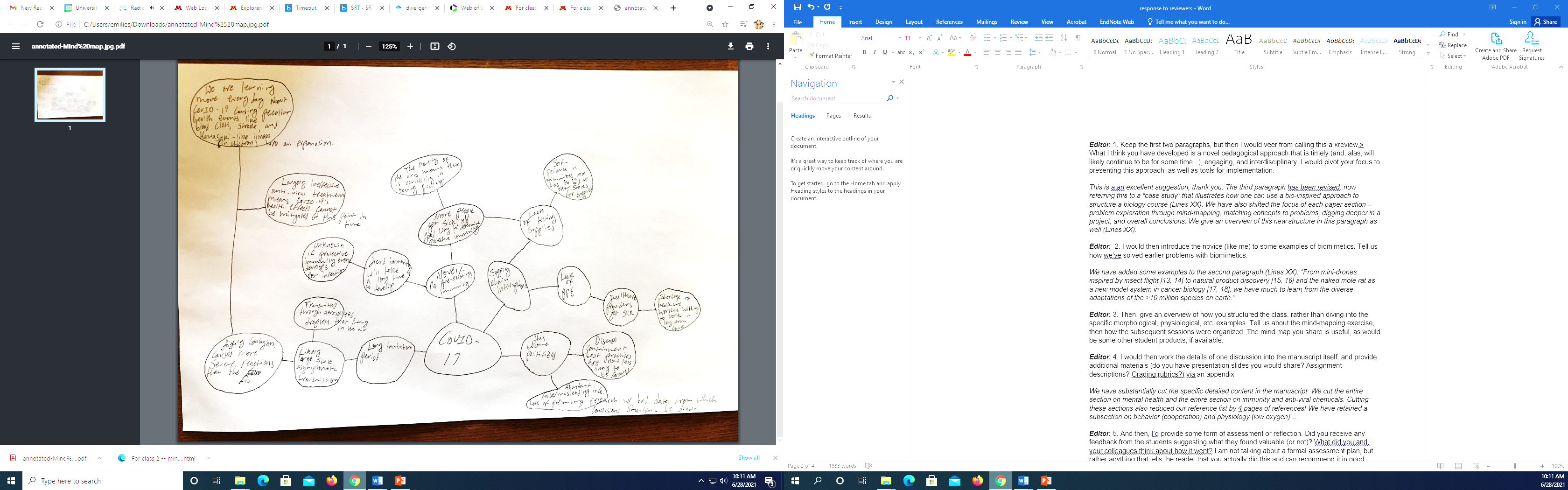


**2. Brainstorming and follow-up with scientific literature (for class 3)**. *Prompt:*

Without looking up anything online, brainstorm a list of as many organisms you can think of where “filtration” may be a relevant function. Please list out all of the systems. There are two papers posted on “how filtration works” in one system. Skim these papers only (maybe 5 minutes each) and write an honest reaction (2-3 sentences only). What strikes you about these studies investigating how a biological filtration system works?

**Sample response:**

1. *The mucous membrane in mouth, nose, and respiratory system*
2. *Kidneys*
3. *Fish gills? Maybe?*
4. *Plant roots*

*This was my first time learning about flamingos or bills on any type of bird. I did not know that bills had filtration systems before reading this paper. I found it interesting that two similar birds can have bills that filter in different ways, allowing them to eat from the same water source but not have to compete for food. I have studied human head and neck anatomy as part of my studies of speech and swallowing, but I know very little about the head or neck anatomy of other animals. It was interesting to look at how the tongue and bill are designed to filter specific sizes of seeds and larvae for the flamingo. I also found it interesting that the tongue of flamingoes has spikes that direct the food down the digestive tract. Reading about the filtration feeding system in flamingoes made me think about how specialized the head and neck anatomy of each species is for eating specific foods.*

**3. A “functional” approach to brainstorming biological systems (for class 5).** *Prompt*: Choose one or two "functions" associated with toilet paper (as you think of it as a covid-19 related problem). What environments or locations in nature come to mind as places to look for biological systems related to this function? If environments don't come easily to mind, think of biological systems first and then list out where you might find them.

**Sample response:**

*--Animals hoarding food for winter reminds me of us hoarding toilet paper during the start of the pandemic: Rodents, Hamsters, Squirrels, Birds, Woodpeckers, Do they gather more than they need?*

*--Absorption: Leaves, Wetlands, deserts*

*--Cleaning: Self-cleaning*

**4. A “divergent systems” approach to brainstorming biological systems (for class 7):** *Prompt:* Focal problem: *oxygen delivery and breathing/respiration.* Make a list of different biological systems/traits that would allow you to come at this problem in different ways. List out the systems/traits and write a few sentences about how you came up with this list.

**Sample response:**

- *Photosynthesizing plant leaves producing oxygen through the conversion of light and CO2.*
- *Fish gills that allow for oxygen from water to diffuse through blood vessels*
- *Flatworm gas exchange via oxygen diffusion through outer membrane.*
- *Insect tracheal system which directly supplies oxygen and facilities CO2 regulation.*
- *Segmented avian respiratory systems comprised of three components--could an external respiratory device be modeled after one of these systems (either in birds or in any of the other systems included here) to provide for an external, temporary lung that not only allows for mechanical respiration, but facilitates mechanical O2 diffusion and CO2 gas exchange?*
- *Some carp and turtles have adapted to survive in anoxic conditions. The Crucian carp is active in anoxic conditions for long periods of time at relatively cool temperatures of 32 deg. F; its hemoglobin is very good at binding to oxygen molecules, and its ATP-dependent processes switch from reliance on aerobic ATP to anaerobic ATP when conditions become more hypoxic--maintaining metabolism levels. The issue with respiration seems to be two fold: physiological impairments of the respiratory system needing supplementation, and--perhaps more importantly--ensuring survival in hypoxic conditions when the respiratory system fails/falters. Quality of respiration needs to be proportional to metabolic activity; is it possible to use external measures to reduce cellular metabolic activity proportional to decreased respiratory quality while still maintaining homeostasis and preventing organ failure/hypoxic brain damage?*
- *Aerial roots of mangroves used to obtain oxygen from air for oxygen provision to roots that are often in hypoxic/anoxic soil conditions. Could this also be a model from facilitating oxygenation and gas exchange?*

*I came up with the list by first examining how different organisms respirated. I then thought about breaking the problem down to the more fundamental question of how one survives in hypoxic conditions for a prolonged period of time without system failure. I then began researching animals that were well adapted to such conditions.*

**5. Brainstorming for less obvious problems (for class 11):** *Prompt:* First, brainstorm a list of traits or biological systems you would go to in thinking about the "mental health" problem that has come out of the covid-crisis. How did you come up with this list? Second, skim the paper (the most relevant sections are 2c and 3c). Does this give you any new ideas for systems or traits for a biomimetic approach to pandemic mental health? Or is the relevance unclear?

**Sample response:**

- *Orangutans/Monkeys (very big on touch)*
- *Schools of fish/dolphins*
- *Large groups of animals that travel in packs*
  - *--> separate by which do it for comfort and which do it for protection*

*I looked at biological systems and organisms that normally travel in groups. Interactions between caregivers and offspring within these groups of organisms can tell a lot about the caregiving and developmental support aspects of each group. Analyzing which groups participate in this show a greater need for support from others of their kind, yet those who travel in groups solely for protection can also tell about the effects of the lack of developmental support. I'm struggling a bit in finding a system that can easily be transferred into a biomimetic approach to address the mental health crisis. Most biological systems rely on touch for comfort, yet this is very restricted in the presence of a pandemic. I think understanding how certain animals begin and maintain their relationships without the help of language is important in understanding how to support one another. Looking at other methods in biological systems other than touch could also be helpful in addressing the contactless method of showing developmental support.*

**6. Deeper dives for class project focused on mask design (for class 12).** Prompt: 1. Do a little research into another biological system/trait of interest and write a few sentences about what you learned (does not have to be related to masks and filtration). 2. Draft a paragraph (stream of consciousness with notes to yourself is fine) about the first system/trait you did research into (last week). Post here and/or on the shared google doc.

**Sample response:**

*Problem: Masks cover the mouth and make communication difficult for individuals with hearing loss, ASD, and social communication disorders.*

*Solution: transparent masks*

*Question 1: What material is transparent, allows for oxygen exchange, and is hydrophobic?*

*Universal mask-wearing has been identified as our best approach to preventing the spread of COVID-19. This simple solution to the COVID-19 pandemic causes increased communication challenges for some individuals. People with hearing loss, ASD, and social communication disorders can have a hard time communicating when they cannot see the mouth of the individual that they are talking to. Creating a transparent mask proves a difficult task because most transparent materials, such as glass and plastics, do not allow air to pass through and fog up when put in front of one’s mouth.*

*It is essential to understand the difference between transparent and translucent when searching for a solution for these masks. Translucent materials let light through, while transparent materials let light through and allow for the formation of an image. The material desired for the mask would need to be transparent in order to allow for better communication outcomes. Transparency is seen in many organisms that live in water, but it is rare in land-dwelling organisms. Some moths and butterflies have transparent wings. Wings of butterflies have hydrophobic properties. The hydrophobicity comes primarily from the scales that are on the wings. Transparent and translucent butterfly wings have fewer scales or modified scales that allow for low-reflectance. Transparent wings are less hydrophobic than other butterfly wings, but they still have hydrophobic properties. This hydrophobicity allows for self-cleaning and durability. The butterfly wings are hydrophobic so that water and dirt are repelled from the wings so that the butterfly stays light and can fly. This hydrophobicity of the butterfly wing would be beneficial in masks, because it would repel aqueous substances from the individual’s airway, protecting individuals from virus particles.*

*The glasswing butterfly’s wings have both transparency and hydrophobicity. Often we look at nature to find patterns, but the transparency of the glasswing butterfly’s wings is due to the seemingly random size and placement of nanostructures on the wings. The Nanostructures are randomly scattered across the glasswing butterfly’s wings, providing anti-reflective properties, and allowing rays of light to pass through (Siddique et al., 2015).*

*Arias, M., Mappes, J., Desbois, C., Gordon, S., McClure, M., Elias, M., ... & Gomez, D. (2019). Transparency reduces predator detection in mimetic clearwing butterflies. Functional Ecology, 33(6), 1110-1119.*

*References*

*Bixler, G. D., & Bhushan, B. (2012). Bioinspired rice leaf and butterfly wing surface structures combining shark skin and lotus effects. Soft matter, 8(44), 11271-11284.*

*Goodwyn, P. P., Maezono, Y., Hosoda, N., & Fujisaki, K. (2009). Waterproof and translucent wings at the same time: problems and solutions in butterflies. Naturwissenschaften, 96(7), 781-787.*

*Hernández-Chavarría, F., Hernández, A., & Sittenfeld, A. (2004). The" windows", scales, and bristles of the tropical moth Rothschildia lebeau (Lepidoptera: Saturniidae). Revista de biología tropical, 52(4), 919-926.*

*Johnsen, S. (2001). Hidden in plain sight: the ecology and physiology of organismal transparency. The Biological Bulletin, 201(3), 301-318.*

*Johnsen, S., & Widder, E. A. (1999). The physical basis of transparency in biological tissue: ultrastructure and the minimization of light scattering. Journal of theoretical biology, 199(2), 181-198.*

*Siddique, R. H., Gomard, G., & Hölscher, H. (2015). The role of random nanostructures for the omnidirectional anti-reflection properties of the glasswing butterfly. Nature communications, 6(1), 1-8.*

*Wanasekara, N. D., & Chalivendra, V. B. (2011). Role of surface roughness on wettability and coefficient of restitution in butterfly wings. Soft Matter, 7(2), 373-379.*

*https://www.businessinsider.com/crazy-nano-structures-make-glasswing-butterfly-wings-clear-2015-5?fbclid=IwAR2ZlHCncxevQRGRHGyzzsryBF_Yi57qFUO0npltDwBER3ncs_SPlOirG00 (Links to an external site.)*

*https://www.chemistryworld.com/features/superhydrophobic-materials-from-nature/3010321.article (Links to an external site.)*

*Species to look at for transparency and hydrophobicity:*

*Pellucid hawk moth, Sphingidae, Cephonodes hylas*

*Glasswing butterfly, Nymphalidae, Greta oto*

*Octauius swordtail, Riodinidae, Chorinea octauius*
