# Supplementary material for: Bioinspiration as a method of problem‐based STEM education: A case study with a class structured around the COVID‐19 crisis
Source: Ecol Evol. 2021 Aug 25;11(23):16374–86. doi: 10.1002/ece3.8044 (PMC8646331; doi:10.1002/ece3.8044)

Class 5: selective environments (and toilet paper)

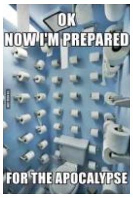

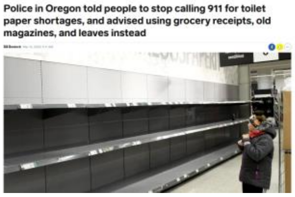
Police in Oregon told people to stop calling 911 for toilet paper shortages, and advised using grocery receipts, old magazines, and leaves instead

What functions or sub-problems come to mind around toilet paper shortages?

- Toilet paper shortage
  - Other toilet/cleaning Products (diapers)
  - Absorption – better options?
  - Cleaning – better options?
  - Why don't animals wipe?
  - Buy-ups and price gauging
  - Home vs business supply chains
  - Panic buying & hoarding

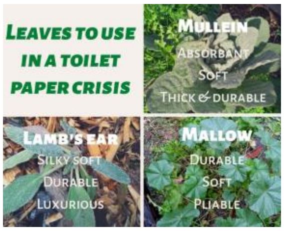

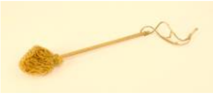

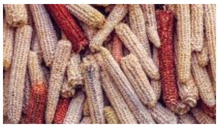

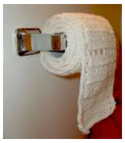

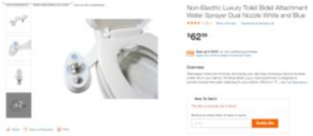

Absorption: *molecules/ions enter a bulk phase, e.g., a solid material*
-Absorption of <u>liquid</u>… vs light, heat, sound

What environments or biological systems do you think of when you think of absorption?

A brief review of the basic mechanism: capillary action in a porous material

Volume liquid absorbed = (area of material wetted)(Sorptivity)sqrt(time)*

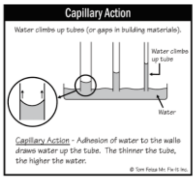

Material	Sorptivity (mm·min$^{-1/2}$)
Aerated concrete	0.50
Gypsum plaster	3.50
Clay brick	1.16
Mortar	0.70
Concrete brick	0.20

What biological <u>systems or traits</u> do you think of when you think of "absorption?"

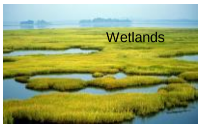
Wetlands

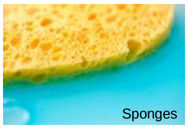
Sponges

Absorption as a byproduct versus a target of natural selection

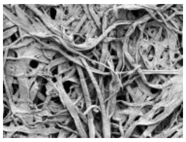
Cellulose fibers - paper towels

What <u>environments</u> do you think of when you think of absorption?

Not enough water

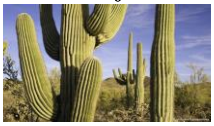

Lots of water

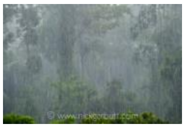

My favorite example… sandgrouse

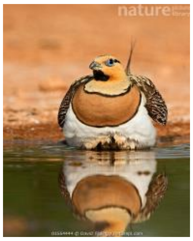

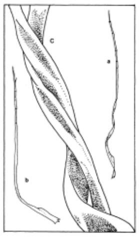

Figure 5. Feather barbules of Namaqua sandgrouse: **a.** dry, **b.** wet, **c.** intertwined bases of adjacent barbules greatly enlarged.

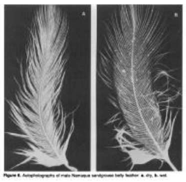

From MacLean 1983 BioScience

Can hold 8g water/g dry feather

https://www.youtube.com/watch?v=Of2Mu_D18I0
https://www.youtube.com/watch?v=ebYrzoPQ4Go

Can we more thoroughly explore the types of environments that "select for a function?"

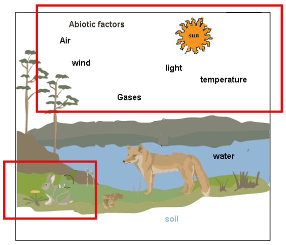

Abiotic factors

Biotic factors

Variation in primary abiotic variables (temperature, water)

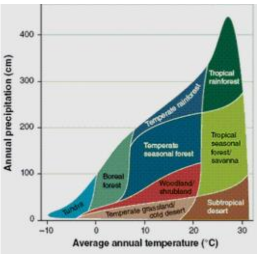

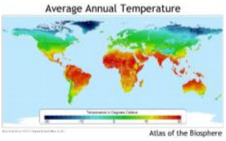

Average Annual Days with precipitation

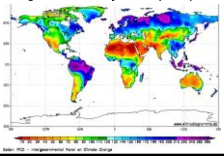

https://en.wikipedia.org/wiki/Biome

Explore life within biomes or geographic regions with variables of interest

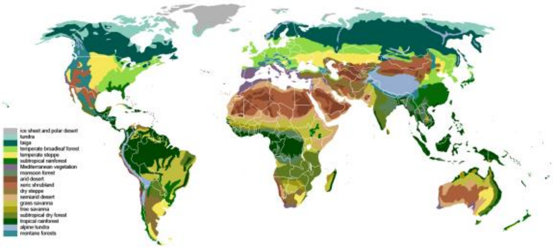

"Minor" abiotic variables may also matter (e.g., winds)

Non-tornadic extreme wind speeds

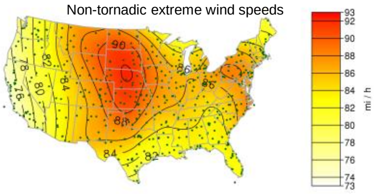

Based on this discussion of abiotic variables, return to the function of "absorption" -- do new ideas or examples come to mind? How would you change a literature search?

867 ecoregions within 14 biomes

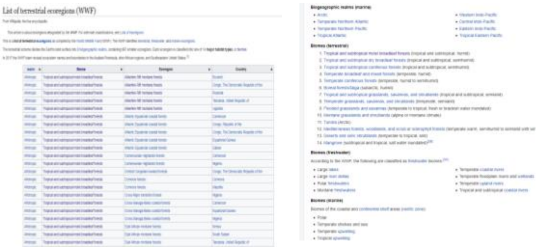

Navigate to one of the 867 ecoregions that might be related to absorption. Why might you want to look at different geographic regions within an ecoregion?

https://en.wikipedia.org/wiki/List_of_terrestrial_ecoregions_(WWF)

https://en.wikipedia.org/wiki/Biome

What about biotic variables?

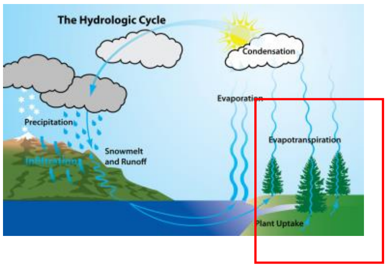

Plants also playing a role in water uptake and likely selection on absorption

Can we make generalizations about spatial variable in biotic variables… sometimes (but it's more difficult)

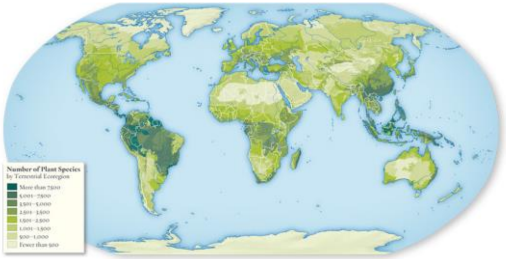

Global patterns of biodiversity
PLANTS

Rate of carbon fixation (net primary productivity) may be a better measure of "plant competition"

Where would you look for "absorption" based on discussion of biotic axes?

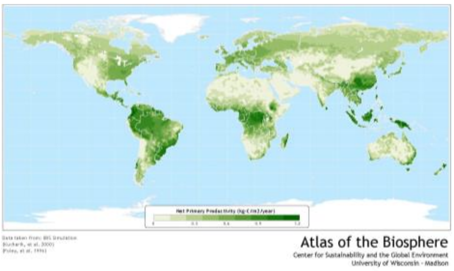

https://nelson.wisc.edu/sage/data-and-models/atlas/maps.php?datasetid=37&includerelatedlinks=1&dataset=37

Complementarity of different desert ecoregions

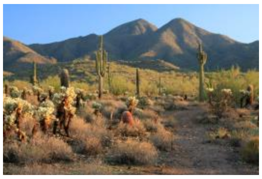

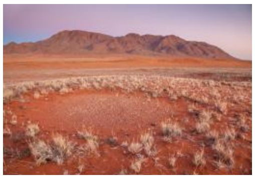

Sonoran Desert
-more biodiverse:
--more biotic competition for water?
--diversity of traits across species?

Namibian Desert
-more extreme low precipitation:
--more extreme adaptations?
--independent origins of traits?

Variation in space/time within an ecoregion/habitat

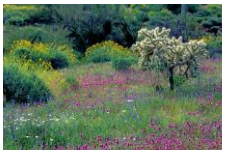

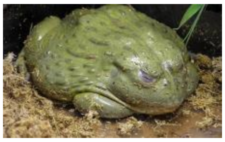

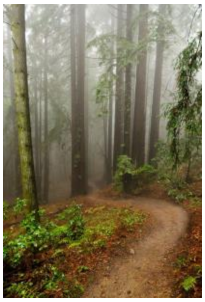

An aside: bioinspired fog harvesters (absorption from gas->liquid)

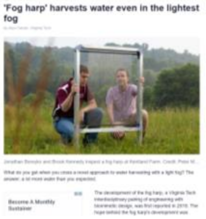

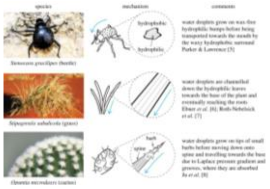

See Shi et al 2020
https://techxplore.com/news/2020-04-fog-harp-harvests-lightest.html

Rev. Guera and Bhushan 2019
Designing bioinspired surfaces for water collection from fog

Back to toilet paper and absorption

- How does a consideration of “selective environments” change how you would explore the space of possible biological systems? Does it?
  - Change your literature search?
  - Change who you would talk to?
  - Change which field guides you might turn to?
  - Spark a different neuron in your brain?

If there is time… take 5 minutes and return to your list of examples for “absorption” – how many more can you add now?

For next class

- We discuss group dynamics and interdisciplinary process in biomimetics
  - Please take brief survey posted on “absorption” systems
- We will discuss the posted reading as example of moving from biology to a design
  - Write a brief paragraph response to the paper
- We will return to discussing our projects
  - Fill out that survey if you haven’t yet…

Remember our discussion of creativity…

- Pilot observations to form hypotheses to test in further classes
  - how to promote group creativity
- Betsy would watch videos after class is over, talk with participants, creativity survey before/after

Creativity research

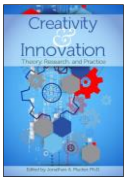

Supplement: Supplementary file 2 — Appendix S2 [file ECE3-11-16374-s002.pdf]
